# Novel Insight into the Composition Differences Between Buffalo and Holstein Milk and Potential Anti-Inflammation and Antioxidant Effect on Caco-2 Cells

**DOI:** 10.3390/foods13233915

**Published:** 2024-12-04

**Authors:** Luyao Wang, Xinyue Hu, Jiaqi Jiang, Dong Wang, Chaobin Qin, Ling Li, Deshun Shi, Qingyou Liu, Jian Wang, Hui Li, Jieping Huang, Zhipeng Li

**Affiliations:** 1 Guangxi Key Laboratory of Animal Reproduction, Breeding and Disease Control, College of Animal Science and Technology, Guangxi University, Nanning 530004, China; wang4183@126.com (L.W.); hxyue2024@163.com (X.H.); jiangjiaqi@st.gxu.edu.cn (J.J.); wangdong20201214@163.com (D.W.); 2018401005@st.gxu.edu.cn (C.Q.); ardsshi@gxu.edu.cn (D.S.); jianwang@gxu.edu.cn (J.W.); lihui3876@163.com (H.L.); 2 Guangxi Key Laboratory of Buffalo Genetics, Reproduction and Breeding, Guangxi Buffalo Research Institute, Chinese Academy of Agricultural Sciences, Nanning 530001, China; lling2010@163.com; 3 Guangdong Provincial Key Laboratory of Animal Molecular Design and Precise Breeding, School of Life Science and Engineering, Foshan University, Foshan 528225, China; qyliu-gene@fosu.edu.cn

**Keywords:** milk, proteome, lipidome, anti-inflammation, antioxidant, Caco-2 cell

## Abstract

Milk is one of the most common sources of nutrients in humans, however, the composition and healthy value of the milk derived from different animals are very different. Here, we systemically compared the protein and lipid profiles and evaluated the anti-inflammation and antioxidant effect of buffalo and Holstein-derived milk on Caco-2 cells. Results showed that 906 proteins and 1899 lipids were identified in the buffalo milk and Holstein milk samples including 161 significantly different proteins (DEPs) and 49 significantly different lipids. The DEPs were mainly enriched in defense response-related terms, while the differential lipids were mainly included in fat digestion and absorption and cholesterol metabolism pathways. In addition, the Caco-2 cells co-cultured with buffalo and Holstein milk components showed significant benefits in being resistant to LPS-induced inflammation stress and H_2_O_2_-induced ROS stress. The qRT-PCR and ELISA results showed that the expression of TNF-α, IL-1β, and IL-6 was significantly lower (*p* < 0.05) in the cells co-cultured with milk components. Further analysis showed that, after H_2_O_2_ treatment, the expression of keap1 and Nrf-2 in the Caco-2 cells co-cultured with milk components was significantly lower (*p* < 0.05). In addition, being co-cultured with milk components significantly decreased the SOD, MDA, CAT, and GSH-Px content (*p* < 0.05) in the Caco-2 cells induced by H_2_O_2_. This study provides a novel insight into the differences in proteins and lipids between buffalo milk and Holstein milk, and a reference understanding of the anti-inflammation and antioxidant effect of the consumption of milk on the intestines.

## 1. Introduction

Milk is one of the most common sources of nutrients in humans due to its abundant fatty acids, proteins, vitamins, minerals, and other nutrients which are beneficial to human health [1,2,3]. However, the content and composition of the nutrients, especially the fatty acids, proteins, and vitamins, in the milk derived from different animals are very different. For example, the contents of fatty acids and protein in buffalo milk are significantly higher than that of cow milk [4,5]. Buffalo milk has higher concentrations of Ca^2+^, Mg^2+^, and P compared to cow milk and contains more vitamin A, vitamin C, vitamin E, and biotin [6,7]. Buffalo milk is known for its higher content of lipids, protein, lactose, and minerals, making it a valuable source of nutrients for human consumption and is suitable for both traditional and industrial dairy product manufacturing [8]. The nutrients not only contribute to the nutritional value of milk, but also play a crucial role in defining market strategies aimed at various consumer groups such as children, nursing mothers, young adults, and the elderly [8].

In addition, nutritional support is also crucial for regulating chronic inflammation and the immune system. For example, saturated fatty acids (SFAs) in milk may promote chronic inflammatory disorders, contributing to the progression of various diseases like inflammatory bowel disease and obesity [9,10]. Meanwhile, positive effects have also been linked to unsaturated fatty acids (UFAs) and conjugated linoleic fatty acids (CLAs) [11]. Several studies have underscored CLA’s role as an anti-atherogenic, anti-inflammatory, antioxidative, and anticarcinogenic agent [12]. Our previous reports have shown that buffalo milk contains more UFAs such as linoleic, linolenic, CLA, eicosapentaenoic acid (EPA), and arachidonic acids than Holstein milk [4,5], suggesting that buffalo milk may provide more value for human health. Moreover, milk is rich in antioxidants including enzymatic antioxidants and non-enzymatic antioxidants such as vitamins, lactoferrin, and casein, which effectively prevent and reduce the formation of free radicals and lipid peroxides [13]. Although milk provides an important source of nutrients, the substance in the milk does not always benefit human health. For example, about 57–65% of people suffer from lactose intolerance, which is caused by a reduction or loss in the activity of lactase in the intestine [14]. Lactose intolerance often leads to abdominal pain, diarrhea, and intestinal inflammation [15]. Therefore, it is of significance to assess the effects of milk on intestinal health.

Human colon adenocarcinoma cells (Caco-2 cells) are widely recognized as a model to simulate the intestinal barrier, facilitating the investigation of metabolite absorption and distribution to distant organs [9,16]. This study compared the protein and lipid profiles of buffalo and Holstein milk. Various components (whole milk, milk whey, or milk fat) from both types of milk were co-cultured with Caco-2 cells to preliminarily assess the anti-inflammatory and antioxidant properties of milk-derived bioactive compounds using an in vitro model. The research provides a novel insight into the protein and lipid differences between buffalo and Holstein milk as well as their potential effects on reducing inflammation and oxidative stress in Caco-2 cells.

## 2. Materials and Methods

### 2.1. Experimental Animals and Sampling

The buffalo milk (a pooled sample from 30 Murrah buffaloes, 5–8 years old, during mid-lactation) and Holstein milk (a pooled sample from 30 cows, 5–8 years old, during mid-lactation) were obtained from two local farms in Nanning, China. Fresh buffalo milk and Holstein milk were collected using an automatic milking machine, respectively. All milk samples were filtered using sterile gauze immediately and transported to the laboratory at 4 °C. Milk samples were pasteurized using domestic pasteurization equipment before cell co-culture. Milk samples for proteome and lipidome analysis were frozen immediately in liquid nitrogen and stored at −80 °C until further use. The routine composition of milk was assessed with a versatile dairy product analyzer (MilkoScan FT-120, FOSS Electric A/S, Hillerod, Denmark). The milk was centrifuged in a centrifugal machine (8000 r/min) at 4 °C. The milk fat was separated in the upper layer, and the milk whey was collected in the lower layer.

### 2.2. Proteome Analysis of the Buffalo and Holstein Milk

Milk samples were centrifugated (4200 r/m) at 4 °C for 20 min and the upper layer was discarded. Protein enzymatic hydrolysis was conducted following the methods outlined in a previous study [17]. Briefly, each sample (200 μg) was supplemented with 8 M UA and incubated at room temperature for 1 h. Subsequently, 100 mM dithiothreitol (DTT) was added and the mixture was incubated again at room temperature for another hour. Iodoacetamide (IAA) buffer (50 mM IAA in UA), 100 mM NH_4_HCO_3_ solution, Trypsin buffer (4 μg Trypsin in 40 μL 25 mM NH_4_HCO_3_), trifluoroacetic acid (TFA), and MCX (Oasis^®^ MCX uElution Plate 30 μm) were sequentially added to facilitate protein extraction. The protein samples were then analyzed using a high-performance liquid chromatography system. The column (Thermo scientific EASY Column, 10 cm, ID75 μm, 3 μm, C18-A2) was pre-balanced with 95% aqueous solution containing 0.1% formic acid (buffer A). The samples were automatically injected into the system and passed through the analysis column at 300 nL/min. Subsequently, the separated samples were entered into a timsTOF Pro mass spectrometer with the following specific parameter settings: ion source voltage (1.5 kV), parallel cumulative sequence (PASEF) scanning interval (100–1700 m/z), time window (1.17 s). To reduce the probability of repeated scanning, the dynamic exclusion time of secondary spectra (0 < z <5) was 24 s. Andromeda was used to analyze the MS data, and the Andromeda score and peptide score were used to evaluate the quality of each data. Peptide and protein FDR ≤ 0.01 were used as the criteria for qualitative analysis. Significance was defined as |Log2(Fold Change)| > 1 and a *p* value < 0.05. Gene Ontology (GO) terms were located and the sequences were annotated by Bblast2GO (v6.0) software. KEGG pathway annotations were performed using KEGG Automatic Annotation Server (http://www.genome.jp/kegg/kaas/, accessed on 16 October 2024) on the target protein set.

### 2.3. Lipidome Analysis of the Buffalo and Holstein Milk Based on LC-MS

The two-phase protocol was used to extract lipids from the milk samples based on previous reports [18,19]. In brief, 750 μL of MeOH:MTBE (1:3) solution containing internal standards (1 mg/L) was added to each sample, followed by vortexing for 1 min and sonication for 15 min in an ice-cooled bath. The samples were then incubated at 4 °C for 30 min and sonicated again. After centrifugation at 14,000× *g* for 10 min at 4 °C, the upper phase was collected, transferred to a new tube, and dried under nitrogen flow. Before mass spectrometry, 200 μL of isopropyl alcohol/acetonitrile (9:1) solution was added to the dried residue to redissolve the lipids. The compound solution was transferred to a new tube and centrifuge (10 °C, 14,000 r/min) for 15 min to remove any possible sediments. Finally, the supernatant was analyzed by mass spectrometry. Sample pools were made by mixing 10 μL of each sample and diluting 1:20 with isopropyl alcohol/acetonitrile (3:1) solution. For each sample, 3 μL was injected into a reversed-phase CSH C18 column (100 mm × 2.1 mm, 1.7 μm diameter particles, Waters) coupled to a Vanguard pre-column with the same dimensions, using a Waters Acquity UPLC system (Waters, Manchester, UK). The mobile phases were composed of acetonitrile and water (6:4) containing 10 mM ammonium acetate and 0.1% formic acid (Buffer A), and acetonitrile/isopropanol (7:3, v:v) containing 10 mM ammonium acetate and 0.1% formic acid (Buffer B). The gradient separation was performed as follows: 3 min at 40% B, followed by a 10 min linear gradient from 40% to 75% B, and then a 6 min linear gradient from 75% B to 100% B. After 5 min wash-in with 100% B, the column was re-equilibrated with 40% B. Mass spectra were acquired in both positive and negative modes using a heated electrospray ionization source (ESI) with a Bruker Impact II QTOF mass spectrometer (Bruker Daltonics, Bremen, Germany). Raw data were automatically calibrated using internal standards, and LipidSearch 4.0 software (Thermo Fisher Scientific, San Jose, CA, USA) was employed for free fatty acid identification [20]. In the adducts panel, –H and –CH_3_COO were selected for negative polarity, and ^+^NH_4_ was selected for positive polarity. The free fatty acids were purified using the top-ranked filter, with minimal isomer peaks identified by the primary node filter and based on fatty acid priority. The parent and product ion tolerance was 5 ppm, and the production threshold was 5%. Lipid feature intensities were normalized via upper quartile normalization. |Log2 (Fold Change)| > 1 and a *p* value < 0.05 were deemed statistically significant.

### 2.4. Co-Culture of the Caco-2 Cells with Milk and Milk Components and LPS/H_2_O_2_ Treatment

The Caco-2 cell lines (purchased from EK-Bioscience, Shanghai, China) were maintained in high glucose DMEM (Gibco, Waltham, MA, USA) supplemented with 20% fetal bovine serum (FBS, Gibco, Waltham, MA, USA), 1% L-glutamine (Sigma-Aldrich, St. Louis, MO, USA), 100 U/mL penicillin/streptomycin (Sigma-Aldrich, St. Louis, MO, USA), 1% non-essential amino acids (NEAA) (Sigma-Aldrich, St. Louis, MO, USA), and 1% sodium pyruvate (Sigma-Aldrich, St. Louis, MO, USA) at 37 °C with 5% CO_2_ in a cell incubator (Thermo Fisher Scientific, Waltham, MA, USA). Caco-2 cells were inoculated in cell culture dishes and cultured overnight. Then, buffalo milk (defined as BM), buffalo milk fat (defined as BMF), buffalo milk whey (defined as BMW), Holstein milk (defined as HM), Holstein milk fat (defined as HMF), and Holstein milk whey (defined as HMW) were mixed with the DMEM culture medium (1:9, *v*/*v*), respectively. The Caco-2 cells were co-cultured with the milk and milk components using the mixed medium for 4 h, and then the mixed medium was replaced with a complete DMEM culture medium. When the cell density reached 80%, the culture medium was refreshed and supplemented with 1 µM LPS (Beyotime Biotechnology, Shanghai, China) or H_2_O_2_ to induce inflammation or reactive oxygen species (ROS), respectively. The cells were further cultured for 24 h (LPS treatment) or 1 h (H_2_O_2_ treatment) and collected for further detection. The Caco-2 cells continuously cultured with a fully supplemented DMEM culture medium were used as the negative control (defined as NC). The time to change the medium was consistent with the mixed medium. The Caco-2 cells cultured with a fully supplemented DMEM culture medium and treated with LPS or H_2_O_2_ were used as the positive control (defined as PC) (Table 1).

### 2.5. RNA Extraction, cDNA Synthesis, and qRT-PCR Analysis

The Caco-2 cells of each group were collected, and the total RNA was extracted with TRIzol. Briefly, 1 mL of TRIzol™ LS reagent (Invitrogen Life Technologies Inc., Carlsbad, CA, USA) was added to the cell pellet and mixed thoroughly. Chloroform (Sigma-Aldrich, St. Louis, MO, USA) was added to separate the aqueous phase, followed by the addition of isopropanol (Sigma-Aldrich, MO, USA) for RNA precipitation. Then, RNA was washed twice with 75% ethanol and resuspended in the DEPC-treated water (Beyotime, Shanghai, China). The concentration and integrity of the RNA were evaluated with a NanoDrop 2000 spectrophotometer (Thermo Fisher Scientific, Waltham, MA, USA) and agarose gel electrophoresis. Genomic DNA was removed using DNase treatment, and first-strand cDNA was synthesized using the HiScript^®^ III 1st Strand cDNA Synthesis Kit (Vazyme Biotech Co. Ltd., Nanjing, China). The single-strand cDNA was kept at −20 °C for future use. qRT-PCR primers were designed with Oligo 7.0 (Table S1). qRT-PCR was conducted using SYBR qPCR Master Mix (Vazyme Biotech Co. Ltd., Nanjing, China) according to the manufacturer’s protocol. A fluorescence ratio PCR instrument (Roche, Shanghai, China) was used to collect the fluorescence data. At least three biological and technical replicates were conducted for each experiment. The gene expression levels were calculated using the 2−∆∆CT method, with GAPDH employed as the reference gene.

### 2.6. Anti-Inflammation and Antioxidant Properties Detection of the Caco-2 Cells

The CCK-8 (Beyotime Biotechnology, Shanghai, China) assay was used to detect the cell viability, the ELISA (Jiubang Biotechnology Co. Ltd., Quanzhou, China) assay was used to detect the content of inflammatory factor in Caco-2 cells, and the OD450 was measured using a Tecan Sunrise. The contents of MDA, SOD, CAT, and GSH-Px in the Caco-2 cells were further measured using a detection kit (Solarbio, Beijing, China) according to the manufacturer’s instructions.

### 2.7. Flow Cytometry

The Caco-2 cells were cultured in 6-well plates until they reached approximately 80% confluence. Then, 1 µM H_2_O_2_ was added and incubated for 1 h to induce the reactive oxygen species (ROS). After incubation, the cell layer was washed three times with pre-warmed PBS to remove any residual H_2_O_2_. The cells were detached from the plate using 0.5% trypsin-EDTA and transferred to a centrifuge tube. They were then centrifuged at 500× *g* for 5 min. The cell pellet was resuspended in flow cytometry standard (FCS) buffer and transferred to a flow cytometry tube. Quantification and analysis were performed using DCFH-DA (Beyotime Biotechnology, Shanghai, China). Data were analyzed using FlowJo software (v10.6.2).

### 2.8. Statistical Analysis

The data were analyzed using analysis of variance (ANOVA) followed by Duncan’s multiple range test to assess differences in both the fatty acid content and gene expression. SPSS version 23.0 was used for the analysis. Results were presented as the mean ± SEM, with statistical significance set at *p* < 0.05.

## 3. Results

### 3.1. Routine Analysis of Milk Composition

The routine milk composition of Holstein cow and buffalo was assessed. Results revealed significantly higher levels of fat, protein, total solids, and solid-not-fat (SNF) in buffalo milk compared to cow milk (*p* < 0.05). However, no notable difference was observed in lactose content between buffalo milk and cow milk (Table 2).

### 3.2. Proteome Analysis of the Buffalo and Holstein Milk

**Figure 1:**
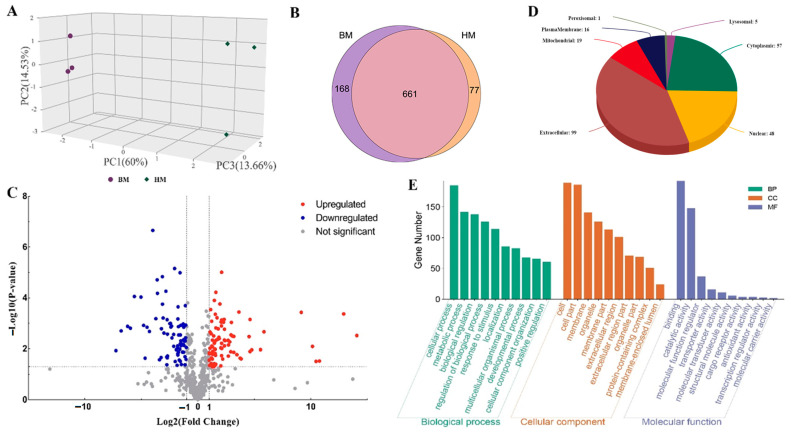
Proteome analysis of the buffalo and Holstein milk. (**A**) Principal coordinates (PCoA) analysis of data from different milk samples. (**B**) Identification of proteins in buffalo milk and Holstein milk. (**C**) Identification of differentially expressed proteins (DEPs) in the buffalo milk and Holstein milk. (**D**) Subcellular localization analysis of the DEPs. (**E**) GO analysis of the DEPs.

We obtained 241,939 peaks from the samples, among which 3226 unique peptides and 906 proteins were identified. Principal coordinates analysis (PCoA) demonstrated distinguishable sample clusters between buffalo milk and Holstein milk (Figure 1A). Among the identified proteins, 661 proteins were found in both the buffalo milk and Holstein milk, 77 were found only in the buffalo milk, and 168 were found only in the Holstein milk (Figure 1B). Further analysis showed that the contents of 161 proteins were significantly different (DEPs) in buffalo milk and Holstein milk including 85 significantly higher and 76 significantly lower proteins in the buffalo milk (Figure 1C). Subcellular localization analysis showed the distribution of these proteins in the cell including the nucleus (48), cytoplasm (57), mitochondria (19), and extracellular regions (99) (Figure 1D). Further GO analysis showed that the DEPs were mainly enriched in “Biological Process” such as defense response to other organisms, “Molecular Function” including endopeptidase regulator activity, endopeptidase inhibitor activity, and hydrolase activity acting on ester bonds (Figure 1E).

### 3.3. Lipidome Analysis of the Buffalo and Holstein Milk Based on LC-MS

**Figure 2:**
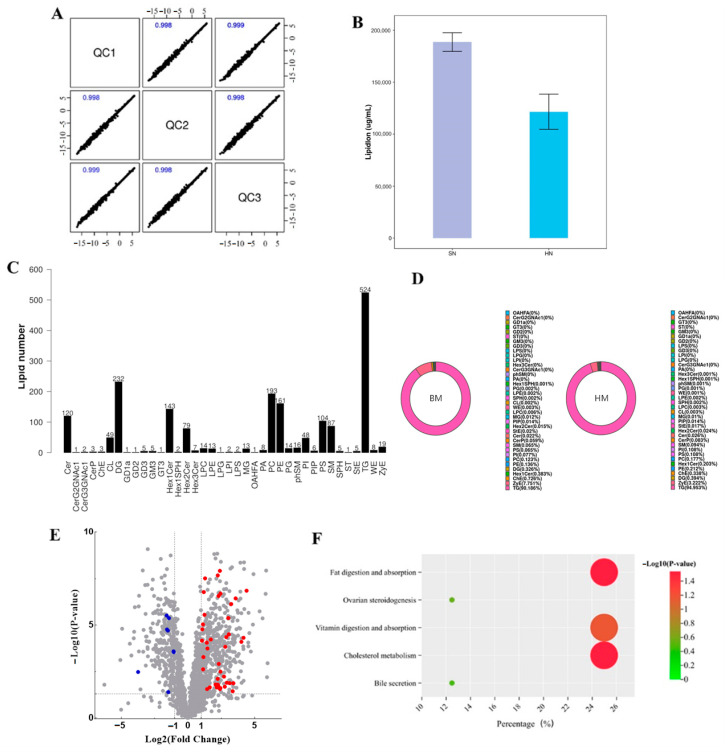
Lipidome analysis of the buffalo and Holstein milk. (**A**) Correlation analysis of QC samples. (**B**) Analysis of total lipid in the milk samples. (**C**) Statistics of lipid subclasses and molecular numbers in the milk samples. (**D**) The proportion of lipid subclasses in different milk samples. (**E**) Differential analysis of the lipids. (**F**) Pathway enrichment analysis of the differential lipids.

Pearson correlation analysis was used to evaluate the correlation between the QC samples. The results demonstrated that the correlation coefficients among all QC samples exceeded 0.9, indicating good repeatability of the detection (Figure 2A). All of the quantifiable lipid molecules in milk samples from the buffalo and Holstein cows were counted, and the total content of lipids in the buffalo milk samples was significantly higher than that in the Holstein milk samples (Figure 2B). A total of 38 lipid subclasses and 1899 lipid molecules including TG, DG, PC, PE, etc. were identified in the buffalo milk and Holstein milk samples, and these lipid subclasses and molecules were shared in all milk samples (Figure 2C,D). Triglyceride (TG) was found to contain the largest number of lipids in both the buffalo and Holstein milk (Figure 2C,D). Further analysis showed that the content of 49 lipids was significantly different between the buffalo and Holstein milk including 41 upregulated lipids and 8 downregulated lipids in the buffalo milk (Figure 2E). Enrichment analysis showed that the main participating pathways of differential lipids included fat digestion and absorption, and cholesterol metabolism (Figure 2F), suggesting that buffalo showed better ability in lipid metabolism regulation than Holstein milk.

### 3.4. Anti-Inflammatory Effect of Buffalo and Holstein Milk Components on Caco-2 Cells

**Figure 3:**
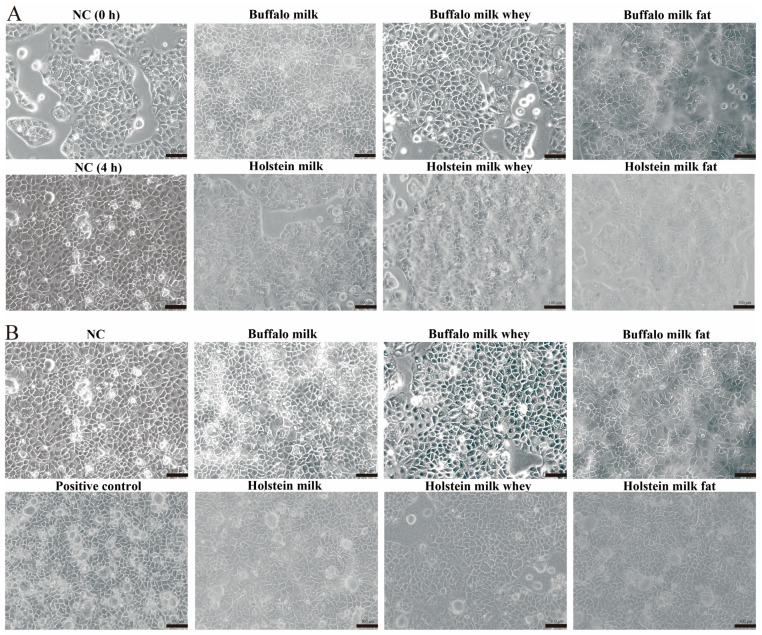
Anti-inflammatory effect of the buffalo and Holstein milk components on Caco-2 cells. (**A**) Caco-2 cells co-cultured with buffalo and Holstein milk components. (**B**) Caco-2 cells treated with LPS (after co-culturing with buffalo and Holstein milk components).

**Figure 4:**
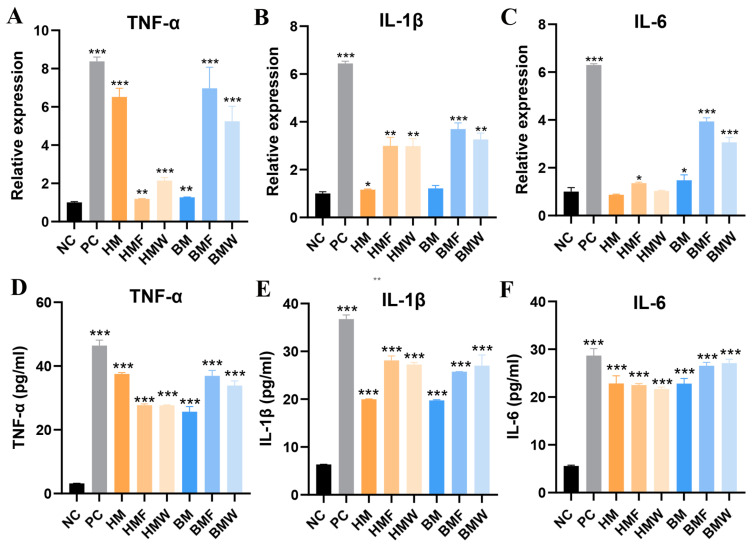
qRT-PCR detection of TNF-α (**A**), IL-1β (**B**), and IL-6 (**C**) in the Caco-2 cells treated with LPS. ELISA detection of TNF-α (**D**), IL-1β (**E**), and IL-6 (**F**) in the Caco-2 cells treated with LPS. * *p* < 0.05, ** *p* < 0.01, *** *p* < 0.001.

Buffalo and Holstein milk components were co-cultured with Caco-2 cells to reveal the anti-inflammatory effect. Results showed that compared to the cells in the NC group, the morphology and integrity of the cells co-cultured with the buffalo and Holstein milk components were even better (Figure 3A). After LPS treatment, the cells in the PC group showed severe vacuolation and fragmentation, while the cells co-cultured with the buffalo and Holstein milk components still showed an intact morphology and integrity (Figure 3B). Further CCK-8 detection showed that Caco-2 cells co-cultured with milk components significantly benefitted the cell viability (*p* < 0.05), suggesting that the components in milk can help Caco-2 cells resistant to LPS-induced inflammation stress (Figure S1). qRT-PCR was conducted to detect the expression of inflammatory factors in cells treated with LPS. Results showed that the expression of *TNF-α* in all groups was significantly improved (*p* < 0.01, Figure 4A). The expression of *IL-1β* in the PC, HMW, BMF, and BMW groups was significantly improved (*p* < 0.01), while the BM group was not significantly altered (Figure 4B). The expression of *IL-6* in the PC, HMF, BM, BMF, and BMW groups was significantly improved (*p* < 0.05), while that in the other groups was not significantly altered (Figure 4C). The concentrations of TNF-α, IL-1β, and IL-6 in the cells were assessed using the ELISA assay. Results indicated that the TNF-α, IL-1β, and IL-6 levels were significantly elevated in all of the LPS-treated groups (*p* < 0.05) (Figure 4D–F). However, the concentrations of TNF-α, IL-1β, and IL-6 in the cells co-cultured with milk components were significantly lower compared to the PC group (*p* < 0.05) (Figure 4D–F), which again suggest that milk and milk components can help Caco-2 cells resistant to LPS-induced inflammation stress. It is noteworthy that no significant difference was found between the co-culture with buffalo milk and Holstein milk.

### 3.5. Antioxidant Effect of Buffalo and Holstein Milk Components on Caco-2 Cells

**Figure 5:**
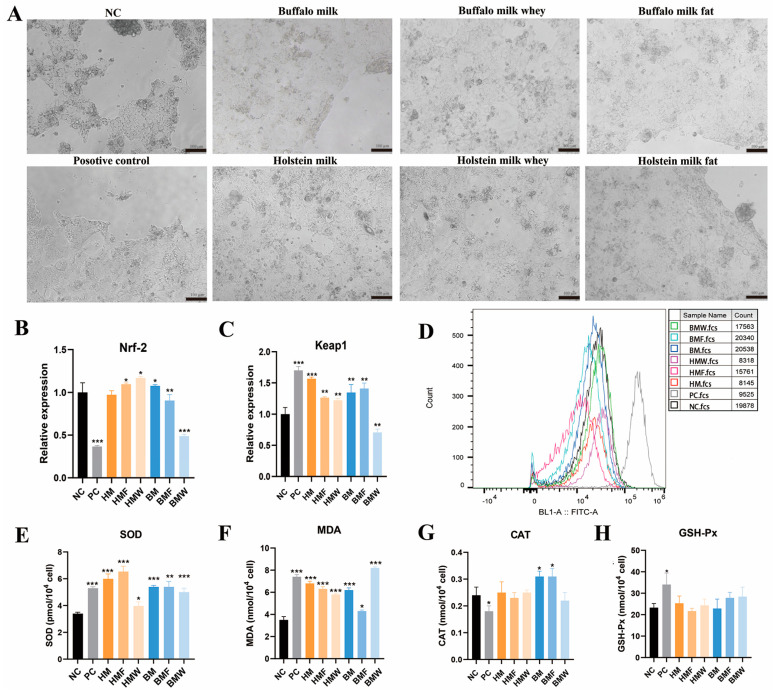
Antioxidant effect of buffalo and Holstein milk components on Caco-2 cells. (**A**) Caco-2 cells were treated with H_2_O_2_ (after co-cultured with buffalo and Holstein milk components). (**B**) qRT-PCR detection of Nrf-2 in Caco-2 cells treated with H_2_O_2_. (**C**) qRT-PCR detection of keap1 in Caco-2 cells treated with H_2_O_2_. (**D**) Flow cytometry detection of ROS in Caco-2 cells treated with H_2_O_2_. (**E**) SOD content in Caco-2 cells treated with H_2_O_2_. (**F**) MDA content in Caco-2 cells treated with H_2_O_2_. (**G**) CAT content in Caco-2 cells treated with H_2_O_2_. (**H**) GSH-Px content in Caco-2 cells treated with H_2_O_2_. * *p* < 0.05, ** *p* < 0.01, *** *p* < 0.001.

The antioxidant effect of the buffalo and Holstein milk components on Caco-2 cells was also analyzed. Results showed that nearly half of the cells died after H_2_O_2_ treatment, while most of the cells co-cultured with milk components survived (Figure 5A). CCK-8 analysis further confirmed the above result (Figure S2). qRT-PCR was conducted to assess the expression levels of keap1 and Nrf-2 in the H_2_O_2_-treated cells. The results indicated a significant reduction in Nrf-2 expression in the PC and BMW groups (*p* < 0.001), while no significant change was observed in the HM group (Figure 5B). Conversely, the expression of keap1 was significantly elevated in the PC, HM, HMF, HMW, BM, and BMF groups (*p* < 0.01), except for a significant reduction observed in the BMW group (Figure 5C). Flow cytometry was further performed by detecting the DCFH-DA in the cells. Results showed that the cells co-cultured with milk components significantly decreased the ROS content in the Caco-2 cells induced by H_2_O_2_. In addition, the ROS content in the cells co-cultured with buffalo milk and its components was significantly lower than that of Holstein milk, and the buffalo milk fat treatment showed the lowest ROS content (Figure 5D). The contents of SOD, MDA, CAT, and GSH-Px in the cells were further detected. Results showed that the SOD content in the cells of all treatment groups was significantly increased (*p* < 0.05) (Figure 5E). The MDA content in the cells of all treatment groups, except for the BMF group, was significantly increased (*p* < 0.001) (Figure 5F). The CAT content was significantly decreased in the H_2_O_2_ treatment cells (*p* < 0.05), while the BM and BMF groups were significantly increased (*p* < 0.05) but did not alter in the other group (Figure 5G). The GSH-Px content was significantly increased in the H_2_O_2_ treatment cells (*p* < 0.05), and did not alter in the milk component co-cultured cells (Figure 5H). The above results suggest that co-culture with milk or milk components can help Caco-2 cells resistant to H_2_O_2_-induced ROS stress. Additionally, no significant difference in antioxidant effect was found between the co-culture with buffalo milk and Holstein milk.

## 4. Discussion

Milk is one of the main sources of nutrients such as fat and protein for humans, and is consumed by more than six billion people worldwide. Buffalo is recognized as the world’s second-largest dairy animal. Buffalo milk has excellent quality, and the fat and protein contents are significantly higher than Holstein dairy cows, which makes it more suitable for the processing of high-quality milk and dairy products, especially solid dairy products. Protein and lipids are the most important nutrients in milk. Analysis of the types and contents of proteins and lipids in milk is helpful to further understand the nutritional value of milk and provide a reference for the development of high-quality dairy products. In recent years, researchers have conducted numerous studies on proteins in milk. In previous studies, based on label-free proteomics, 133 proteins were identified in the milk of the Binglangjiang buffalo and Dehong buffalo [21], while 95 proteins were identified in Holstein milk [22]. In a separate study, 23 proteins were identified in buffalo milk using shotgun proteomics [23]. In this study, buffalo and Holstein milk were analyzed based on 4D label-free proteomics technology, and 906 proteins were identified, which was far more than that in previous studies, and provided a reference for a further understanding of proteins in milk. Further analysis indicated that the DEPs were predominantly distributed in the nucleus, cytoplasm, mitochondria, and extracellular regions, which is consistent with previous study [24]. Buffalo milk contains more active lysozymes than Holstein milk and is considered to have better antimicrobial properties [25]. In this study, we found that the DEPs were significantly enriched in biological processes such as antimicrobial humoral response, defense response against bacteria, and defense response against other organisms, which was consistent with the previous reports [26,27], suggesting that the immune defense mechanism of buffalo is stronger than that of Holstein. The lipidomic analysis found that the total lipids in the buffalo milk samples were significantly higher than those in the Holstein milk samples, which was consistent with previous findings [28]. Further differential analysis on the content of lipid subclass showed that compared with Holstein milk, ChE, GM3, Hex1Cer, Hex1SPH, LPC, OAHFA, PG, WE, and ZyE were significantly upregulated in buffalo milk, while GD1a and GD3 were significantly downregulated. Differential lipids are predominantly enriched in the pathways of fat digestion and absorption as well as cholesterol metabolism. ChE is a compound formed by the esterification of cholesterol and fatty acids, which is mainly present in the blood as a part of lipoprotein [29]. The main function of ChE is to help distribute and transport cholesterol in the body, which is significantly related to cardiovascular health [30]. Therefore, the higher ChE in buffalo milk indicates that it is beneficial for cardiovascular health. Hex1Cer and Hex1SPH are involved in the sensitivity of apoptosis and growth factor signaling [31]. LPC can enhance the activation of T lymphocytes and the antibody production of B lymphocytes. Meanwhile, LPC has immunomodulatory effects in vivo such as enhancing phagocytosis, promoting the recruitment of immune cells, and inhibiting inflammation caused by endotoxin [32], which may be one of the reasons why buffalo are not susceptible to mastitis. Therefore, revealing the characteristics of proteins and lipids in milk can provide a reference for further understanding the functions of milk in growth and development, immune regulation, and disease prevention as well as provide direction for the research development and production of functional dairy products.

Food–intestine interaction studies have been a focal point in food science and nutrition due to the multifaceted physiological roles of the intestine. Intestinal cell models are widely utilized in such studies due to their simplicity, reliability, and high reproducibility. The human epithelial cell line Caco-2, originally derived from colon carcinoma, has been extensively employed as a model for studying the intestinal epithelial barrier [33]. The Caco-2 cell line is heterogeneous and consists of cells with diverse properties. These cells spontaneously differentiate into a monolayer resembling absorptive enterocytes found in the small intestine, characterized by a brush border layer, tight junctions, the secretion of hydrolases, and the synthesis of carrier transport systems for sugars, amino acids, and drugs [33,34]. Previously, Caco-2 cells were co-cultured with goblet cell-like HT29-MTX cells, resulting in the formation of a functional epithelial barrier. Subsequent incubation with the cholera toxin increased short-circuit currents, indicative of enhanced apical chloride secretion [35]. Moreover, the Caco-2 cell line has been pivotal in investigating the absorption and transport mechanisms of functional foods and drugs across the intestinal epithelium to explore their antioxidant, anticancer, and anti-inflammatory properties. Various studies have highlighted the potential of functional food extracts (FFEs) tested using Caco-2 cells to mitigate inflammation, combat cancer, ROS, and lower cholesterol levels by attenuating local inflammatory signals, reactive oxygen species production, and lipid accumulation [36]. For example, a study utilizing the Caco-2 cell line assessed the absorption rates and mechanisms of AVNs and found significantly lower apparent absorption rates (Papp) for AVN 2c, AVN 2f, and AVN 2p compared to caffeic acid and ferulic acid [37]. Another investigation evaluated the antioxidant and anti-inflammatory effects of four types of commercial milk, along with their total and free lipid fractions, revealing that raw milk exhibited higher antioxidant capabilities compared to rice [9]. In this study, we evaluated the antioxidant and anti-inflammation effect of buffalo and Holstein-derived milk and its’ components on Caco-2 cells and found that co-culture with milk and milk components significantly improved the cell’s resistance to the LPS and H_2_O_2_-induced stress, which provide a novel understanding about milk consumption on human health.

However, although many studies have reported that milk contains diverse substances that have multifunctional activities such as immunomodulatory, anti-inflammatory, antimicrobial, and anticancer properties [38,39], little study has focused on the effect of raw milk on the antioxidant and anti-inflammation effect in human intestine health. The intestine serves as the body’s largest immune organ. Intestinal tight junctions (TJs) and the intestinal mucosa, which include a variety of immune cells, constitute the innate immune system. This system protects the host against antigens, bacteria, and viruses that originate from food in the intestinal cavity [40]. Oxidative inflammation has emerged as a significant health concern, with numerous studies documenting the interdependent relationship between oxidative stress and inflammation [41]. Milk proteins have been found to have multifunctional activities such as immunomodulatory, anti-inflammatory, antimicrobial, and anticancer properties [38,39]. In this study, we found that the cell viability of Caco-2 cells co-cultured with milk components was significantly improved by altering the expression of TNF-α, IL-6, and IL-1β, which is consistent with the report. Nrf-2 can bind to response elements in the promoter regions of a variety of antioxidant genes and initiate gene expression, thereby increasing the levels of antioxidant enzymes and attenuating tissue damage caused by oxidative stress [42]. It has been reported that the antioxidant substances in milk can inhibit the formation and remove the ROS [38]. In the present study, it was found that all components of buffalo milk and Holstein milk were able to reduce oxidative stress induced by H_2_O_2_-induced stress by promoting the expression of *Nrf-2* and suppressing the expression of *keap1*, which is consistent with the report. SOD, CAT, and GSH-Px are important antioxidant enzymes in the pathogenesis of oxygenation stress that can catalyze the reduction of oxygen radicals to hydrogen peroxide, which can be further reduced to water under the catalysis of CAT [43]. In this study, we found that Caco-2 cells co-cultured with milk and milk components significantly reduced the ROS levels by increasing the vitality of SOD, CAT, and GSH-Px, further suggesting that co-culture with milk or milk components can help Caco-2 cells resistant to H_2_O_2_-induced ROS stress.

As discussed above, Caco-2 cells have been widely used to study intestinal absorption, barrier function, and the transport mechanisms of drugs and nutrients in vitro. After a 21-day differentiation process, Caco-2 cells gradually form a mono-layer with polarized structures that resemble the characteristics of intestinal epithelial cells. The differentiated Caco-2 cells exhibited typical features of intestinal epithelial cells such as tight junctions, the formation of microvilli on the surface, and the ability to transport substances, which make Caco-2 cells an ideal model for studying intestinal absorption, toxicological experiments, drug transport, inflammatory responses, and other physiological processes. For example, a previous study analyzed the antioxidant activity of four bioactive peptides derived from milk proteins in differentiated Caco-2 cells and found that all four peptides were effective in maintaining cell viability and resisting induced oxidative stress [44]. Another study used the differentiated Caco-2 cells as a gastrointestinal organoid model to investigate the effects of melatonin on gastrointestinal viral inflammation induced by Poly I:C and found that melatonin induced antiviral effects in Caco-2 cells through the type III IFN pathway [45]. Additionally, a study compared four different phenolic fractions in an LPS-induced differentiated Caco-2/RAW264.7 co-culture model and found that free phenolic compounds reduced inflammation by inhibiting the PI3K/Akt/NF-κB signaling pathway and effectively suppressed LPS-induced intestinal permeability and the loss of tight junction proteins [46]. However, we also found that many studies used undifferentiated Caco-2 cells to investigate the potential effects of nutrients on the intestine in vitro. For example, a study characterized the lipid profiles of four types of commercial milk and compared their importance in regulating the antioxidant capacity and cell viability in response to inflammation stimulation in undifferentiated Caco-2 cells [9]. Another study investigated the ability of bergamot fruit extract (BFE) and its major components to regulate cholesterol levels in undifferentiated Caco-2 and HepG2 cells [13]. Moreover, a study treated undifferentiated Caco-2 cells with varying concentrations of curcumin (Cur) for different durations and assessed the cell proliferation, finding that curcumin inhibited Caco-2 cell proliferation in a dose- and time-dependent manner [47]. Therefore, the scientific validity of using undifferentiated cells needs further discussion. In this study, we used the undifferentiated Caco-2 cells to investigate the potential effects of milk on the intestinal anti-inflammatory and antioxidant properties. However, this approach did not seem to be the best solution for addressing the issue. Following the reviewer’s suggestion, we further explored the physiological characteristics of Caco-2 cells and found that the differentiated Caco-2 cells seemed to be a more accurate model. However, based on the literature we referenced, this study primarily focused on the preliminary investigation of the anti-inflammatory and antioxidant effects of cow’s milk and buffalo milk using undifferentiated cells. Moreover, milk, being a mixture, contains a large amount of macromolecules such as fats, which theoretically cannot directly interact with undifferentiated cells. Therefore, before conducting research, it is necessary to perform in vitro digestion. However, in vitro simulation experiments usually need digestive enzymes and treatment with other substances. Although various digestion methods have been reported in the literature, none can perfectly replicate the in vivo digestive environment, and the experimental results often show considerable variability. Additionally, we observed that many studies used undigested milk or other substances for study [9,48,49]. For example, one study analyzed the lipid profiles of four commercial milks without digestion treatment and co-cultured undifferentiated Caco-2 cells under H_2_O_2_ stimulation to assess LPS-induced inflammatory responses [9]. Another study analyzed the effect of different undigested buffalo milk sources by co-culturing with endothelial cells treated with high glucose [48]. Another study investigated the effects of untreated Lactobacillus bicolor honey extract and its biomarker (Trifolin) on DSS-induced Caco-2 cells and found that treatment with L. bicolor honey extract and Trifolin enhanced the integrity of tight junctions in damaged Caco-2 cells [49]. Based on the above reports and our objectives, the undigested samples were used in this study. All in all, we believe that, to a limited extent, our study sheds some light on the potential anti-inflammatory and antioxidant effects of milk.

## 5. Conclusions

This study constructed the protein and lipid profiles of buffalo and Holstein milk and revealed some significant functional proteins and lipids that were differentially expressed in the two kinds of milk. We further systemically evaluated the anti-inflammation and antioxidant effect of buffalo and Holstein-derived milk and its components by co-culturing with Caco-2 cells and found that co-culture with milk and milk components significantly improved the cells resistant to the LPS and H_2_O_2_-induced stress. Although diverse proteins and lipids were found between the buffalo and Holstein milk, no significant difference in anti-inflammation and antioxidant effect was found between the co-culture with buffalo milk and Holstein milk. This study provides a novel understanding of the differences in the proteins and lipids between buffalo milk and Holstein milk and shows that the consumption of both kinds of milk has the potential benefit as an anti-inflammatory and antioxidant in the intestines.

## Figures and Tables

**Table 1:** Different treatment of the Caco-2 cells in different groups.

	DMEM Culture Medium	Buffalo Milk	Buffalo Milk Fat	Buffalo Milk Whey	Holstein Milk	Holstein Milk Fat	Holstein Milk Whey	LPS (24 h)	H_2_O_2_ (1 h)
NC	+	-	-	-	-	-	-	-	-
PC	+	-	-	-	-	-	-	1 μM	1 μM
BM	+	+	-	-	-	-	-	1 μM	1 μM
BMF	+	-	+	-	-	-	-	1 μM	1 μM
BMW	+	-	-	+	-	-	-	1 μM	1 μM
HM	+	-	-	-	+	-	-	1 μM	1 μM
HMF	+	-	-	-	-	+	-	1 μM	1 μM
HMW	+	-	-	-	-	-	+	1 μM	1 μM

Note: “+” indicates co-culture with the Caco-2 cells (mixed with the DMEM culture medium, 1:9, *v*/*v*). “-” indicates did not add to the culture medium. LPS/H_2_O_2_ treatment was performed independently.

**Table 2:** Routine composition of buffalo milk and Holstein milk.

	Protein %	Fat Content %	Total Solids %	SNF %	Lactose %
Buffalo Milk	5.26 ± 0.76 ^a^	8.37 ± 1.28 ^a^	19.82 ± 2.64 ^a^	10.55 ± 0.4 ^a^	4.98 ± 0.14 ^a^
Cow Milk	3.36 ± 0.37 ^b^	4.01 ± 0.87 ^b^	13.28 ± 1.08 ^b^	8.65 ± 0.7 ^b^	5.10 ± 0.23 ^a^

Note: Results are presented as mean ± SEM. Superscripts a, b indicate significant differences (*p* < 0.05).

## Data Availability

The original contributions presented in the study are included in the article/Supplementary Material, further inquiries can be directed to the corresponding authors.

## Supplementary Materials

The following supporting information can be downloaded at: https://www.mdpi.com/article/10.3390/foods13233915/s1, Figure S1: CCK-8 analysis of the cell viability of Coca-2 cells treated with LPS; Figure S2: CCK-8 analysis of the cell viability of Coca-2 cells treated with H_2_O_2_; Table S1: Primers used in this study.
