# Supplementary material for: Novel Insight into the Composition Differences Between Buffalo and Holstein Milk and Potential Anti-Inflammation and Antioxidant Effect on Caco-2 Cells
Source: Foods. 2024 Dec 4;13(23):3915. doi: 10.3390/foods13233915 (PMC11640712; doi:10.3390/foods13233915)
Supplement: Supplementary file 1 [file foods-13-03915-s001.zip › foods-3291330-supplementary.pdf]

Supplementary materials

Figures

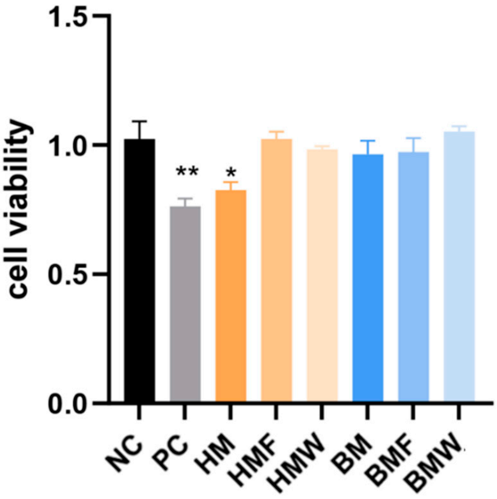

Figure S1 CCK-8 analysis of cell viability of Coca-2 cells treated with LPS.

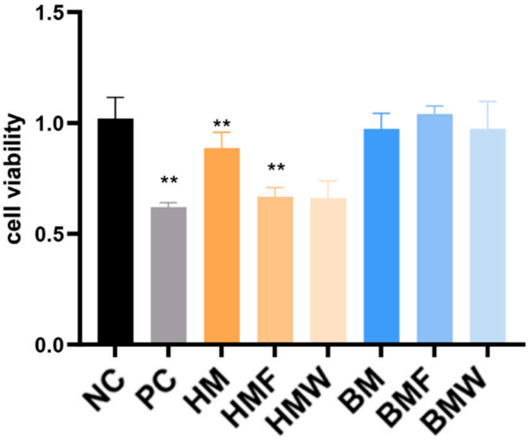

Figure S2 CCK-8 analysis of cell viability of Coca-2 cells treated with H_2O_2.

Tables

Table S1 Primers used in this study

Primer	Sequences (5'-3')	Lengths (bp)
TNF-α	F: TAGCCCATGTTGTAGCAAACC<br>R: ATGAGGTACAGGCCCTCTGAT	136
IL-6	F: CCAGAGCTGTGCAGATGAGT<br>R: AAGTGGCATTGCATCCCTGA	142
IL-1β	F: CCTGAGCTCGCCAGTGAAAT<br>R: GTCGGAGATTCGTAGCTGGA	150
Nrf-2	F: ATCCATTCCTGAGTTACAGTGTCT<br>R: TCTGTCAGTTGGCTTCTGGA	89
Keap1	F: AGACGTGGACTTTCGTAGCC<br>R: CCAGGAACGTGTGACCATCA	111
GAPDH	F: GCACCGTCAAGGCTGAGAAC<br>R: TGGTGAAGACGCCAGTGGA	138
